# Study on the Use of Tobacco Among Male Medical Students in Lucknow, India*

**DOI:** 10.4103/0970-0218.40877

**Published:** 2008-04

**Authors:** Ranjeeta Kumari, Bhola Nath

**Affiliations:** Department of Community Medicine, Sri Laxmi Narayna Institute of Medical Sciences, Pondicherry, *Institution where work is carried: King George Medical College, Lucknow, Uttar Pradesh, India

**Keywords:** Medical student, smoking, tobacco

## Abstract

**Objectives:**

Is use of tobacco a major health problem among medical students? To find out the factors associated with the use of tobacco.

**Materials and Methods:**

A cross-sectional study was done on 250 undergraduate male medical students using a pre-designed, pre-tested questionnaire to study about the problem and various correlates of the tobacco use. Data was collected and analysed using Excel and SPSS software.

**Results:**

Among the tobacco users (28.8%), smoking was found in 87.5% and tobacco chewing in the form of gutka, khaini, gulmanjan (locally available forms of tobacco) in 37.5% as the predominant means of the use of tobacco. The mean age of our sample was 23.5 years. The residential background, i.e., rural or urban, and religion were not significantly associated with the use of tobacco in the present study. Hostellers were found to be more frequent tobacco users as compared to day-scholars. There was a familial aggregation of the use of tobacco. The factor initiating the use of tobacco was usually peer pressure.

**Conclusion:**

Tobacco use is a significant problem among the male medical students and we need to take steps to stop its use by them so as to prevent them from being exposed to its hazardous effects. This will also make their role in the advocacy of the smoking cessation activities more trustworthy.

Use of tobacco is the second major cause of death in the world.(1) Each year, tobacco products kill some 5 million people worldwide and this number is increasing. WHO estimates that, unless current smoking patterns are reversed, tobacco will be responsible for 10 million deaths per year, by the decade 2020-2030, with 70% of them occurring in developing countries. Scientific evidence has been accumulating since the early 1950's and more than 25 diseases are now known or strongly suspected to be causally related to smoking.(2–4)

Teaching about the effects of the use of tobacco and related diseases is essential for the undergraduate medical students, especially to counter the deadly effects of the same. Physicians occupy a key position in this regard, as they are uniquely placed to lead smoking cessation programmes in the community(5); but if the future physicians are themselves entangled in the web of the abuse and dependence of tobacco, then the plight of the smoking cessation programmes can well be imagined.

As medical students, who are usually in their adolescence, progress through the medical school, their behaviour regarding the use of tobacco equals or even exceeds the rates in the non-medical peer groups, despite their knowledge of smoking-related diseases. As such, many researchers have historically investigated tobacco-smoking rates among this demographic group.

As future physicians who will witness the continued burden of smoking-related diseases among their patients, medical students represent a primary target for tobacco-prevention programmes. Therefore, the purpose of the current paper was to evaluate the use of tobacco in the male medical students and to elucidate the factors associated with its use.

## Materials and Methods

### Study design and participants

A cross-sectional study was done among the male medical undergraduate students of King George Medical College, Lucknow, a city in North India. The students were briefed about the purpose of the study. Out of the total male medical students, we could distribute the pretested structured questionnaires to a total of 423 male undergraduate medical students. Participation in the study was voluntary and verbal informed consent was taken from the participating students. Out of the total 423 students, only 265 students had responded, out of which 15 had given incomplete response and were therefore excluded from the study. Thus, 250 completed questionnaires were used for the analysis. Students were not required to write their names to ensure confidentiality and to elicit correct responses from them. The information was collected regarding age, religion, residential background, current place of living, age at initiation, precipitating factor for the initiation, form of product used, pattern of tobacco use and tobacco use in the family. “Ever use” was defined as ‘having used tobacco even once in their lifetime’. “Current use” was defined as ‘having used tobacco at least once in the last 30 days preceding the survey’. “Never use” was defined as ‘having not used tobacco even once in their lifetime’.(6)

### Study period

The survey was conducted from August to September 2005.

### Statistical analysis

The data collected was tabulated, coded and analyzed using Microsoft Excel and SPSS for Windows, version 10.5. Chi-square test and Fishers' exact test were used for evaluating the statistical significance of the association between the independent and the dependent factors. For all the tests, *P*-value <0.05 was considered significant.

## Observation and Results

### Form and pattern of tobacco use

The results in the present study revealed that, out of total students (*n* = 250), 72 (28.8%) were found to be current tobacco users. Among these, 63 students, i.e., 87.5% were found to be consuming tobacco as cigarettes or other forms of smoking, while the use of smokeless tobacco in the form of gutka, khaini, gulmanjan (locally available forms of tobacco) was found in 27, i.e., 37.5% of the total current tobacco users [Table 1]. The mean age of our study subjects was 23.5 ± 1.7 years.

**Table 1:** Form and pattern of use of tobacco

Smoking status	Number	Percentage (95% confidence interval)
Current tobacco users* (72)		
Smoking	63	25.2 (19.9-31.1)
Use of tobacco in any other form	27	10.8 (7.2-15.3)
Non-tobacco users, currently (178)		
Ever users	10	4.0 (1.9-7.2)
Never users	168	67.2 (61.0-73.0)

* Includes multiple responses

### Current tobacco use in the students and its correlates

We also came to a significant conclusion through this study that the residential background of the student, i.e., rural or urban, was not significantly associated with the tobacco use. Another important observation was that religion also had no association with the use of tobacco. The apparent difference in the percentage of tobacco use in Hindus and Muslims was not statistically significant.

The current place of residence was found to significantly affect the use of tobacco. The hostellers (21.8%) were found to be using more tobacco as compared to the day-scholars (11.1%).

The familial aggregation of the tobacco use was also quite evident in the present study, with tobacco use being more common among students belonging to families where tobacco use is prevalent [Table 2].

**Table 2:** Bivariate analysis of current tobacco use in the students

Characteristics of students (*n* = 250)	Tobacco users (72)	Non-tobacco-users (178)	χ^2^-value, *P*-value
Religion			
Hindus (234)	70 (29.9%)	164 (70.1%)	1.45, *P* < 0.229
Muslims (16)	2 (12.5%)	14 (87.5%)	
Current place of residence			
Hostellers (214)	68 (21.8%)	146 (68.2%)	5.449, *P* < 0.019*
Day scholars (36)	4 (11.1%)	32 (88.9%)	
Practice among family members			
Family users (123)	60 (48.8%)	63 (51.2%)	45.24, *P* < 0.00*
No family users (127)	12 (9.4%)	115 (90.6%)	
Family background			
Rural (83)	25 (30.1%)	58 (69.9%)	0.03, *P* < 0.86
Urban (167)	47 (28.1%)	120 (71.9%)	

* *P* < 0.05 statistically significant

An enquiry into the factors leading to the initiation of the use of tobacco revealed that it is mostly initiated due to peer pressure (78%). The other, less important factors responsible were curiosity and the effect of family members.

## Discussion

Various efforts have been made in the direction of assessing the effect of various factors on the smoking behaviour among the medical students in different parts of the world. With the increasing use of the smokeless forms of tobacco as well, it has become important to bring out the data regarding the overall use of tobacco and its various correlates. Therefore, we have tried to find out the overall burden of tobacco use among the male medical students, who may serve as the role model for the patients with respect to the smoking cessation activities.

The proportion of current tobacco users in our study sample was found to be 28.8%, with the smokers constituting 25.2%. The overall prevalence of current use of tobacco in the population above 10 years of age was observed to be 34.4% in Uttar Pradesh (50.0% among males and 9.1% among females), while that of current smoking was 18.0% (27.5% among males and 2.6% among females) in Uttar Pradesh.(7) The percentage of current smoking in our study sample is comparable to that in the general population and is a matter of serious concern. The findings of our study are also comparable to the results of a similar study done in the neighbouring country Pakistan, which revealed a 22% prevalence of smoking among the male medical students.(8) The figures are comparable to our study and reveal that religion, most probably, does not have much effect on the use of tobacco. A report from the study in 15 medical schools from nine Asian countries revealed that the prevalence of daily smoking in males varied from 4 to 11% from first year to final year; of occasional smoking 18 and 24%, respectively,(9) indicating that the use of tobacco does not respect international boundaries. However, the rates of smoking vary from that in our study because this study was spread over nine countries which have a variation in the use of tobacco between them. The figures obtained were a reflection of an average of all the nine countries and are therefore less specific and comparable with our study which is more localized. The corresponding figures of tobacco use in similar studies done in Kerala (14.1%),(10) Orissa (12.4%)(11) and West Bengal (3.2% among the newly admitted medical students)(12) were found to be quite low, which may be due to the various other unidentified factors.

The absence of an association between the residential background and the tobacco use highlights the importance of the spread of the epidemic of tobacco use. Similar observations have been made in the young boys in the general population of Uttar Pradesh(7) and in another similar study on the male medical students in 1989,(13) which may be an indication that the trend of tobacco use is deep-rooted and not a recent one.

The preventive effect of religion and the other cultural factors on the behaviour related to the use of tobacco seems to be losing its impact, with religion having no association with the use of tobacco in the present study. The effect of religion on tobacco use has not been evaluated in other studies and thus we lack the data for comparison.

The preventive effect of parental supervision on the use of tobacco was quite evident in our study and another similar one in Orissa,(11) and in Pakistan(8) with hostellers using more tobacco as compared to day-scholars. The results showing this association are available in quite a few studies done in India, and this factor needs to be explored further.

There was a significant relationship between the presence of a smoker in the family and picking up the habit in the present as well as other studies,(8,13,14) further substantiating the familial aggregation of the tobacco use.

The overwhelming effect of peer pressure on the initiation of tobacco use is a matter of serious concern because it is very difficult to prevent the effect of this factor in an age group which likes the company of their friends as well as is influenced maximally by them, more so while living in a hostel away from their homes. The web of causation of this particular factor is very complicated and it has a direct as well as an indirect and synergistic effect with other factors. Disentangling it would require even more in-depth research.

## Limitation

The prevalence of current tobacco users observed in our study could be an underestimation considering the fact that only 265 students responded out of 423 students. There could be a possibility that users of tobacco would not have participated in the study despite the assurance of maintaining confidentiality of the information provided.

## Conclusion

We took medical students as the focus of our survey, as the approach and credibility of future physicians as treatment providers for smoking - and tobacco-related diseases may be influenced by their smoking habits. The results in our study are discouraging and reveal that the medical knowledge regarding the ill effects of tobacco use has not been able to check its use. We need to reduce the use of tobacco among medical students so that the general public can accept them as their role models in the smoking cessation activities. The social acceptability of tobacco use contradicts the strong health education and health promotion messages discouraging it. This may require legislative steps banning the use of tobacco in the college campus, but more importantly, specific training and counselling of the students on a regular basis to help them overcome the desire to indulge in this deadly habit.
